# Teaching the Atomic Sentence Method for Source-Verified, AI-Assisted Literature Synthesis to Clinical Health Care Professionals: Single-Cohort Feasibility and Acceptability Study

**DOI:** 10.2196/98343

**Published:** 2026-08-27

**Authors:** Sung-Wei Liu, Shao-Yin Chu, Hung-Che Wang, Ming-Shinn Lee

**Affiliations:** 1 Department of Medical Education and Emergency Medicine, Hualien Tzu Chi Hospital, Buddhist Tzu Chi Medical Foundation, Hualien, Taiwan; 2 School of Medicine, Tzu Chi University, Hualien, Taiwan; 3 Department of Medical Education and Pediatrics, Hualien Tzu Chi Hospital, Buddhist Tzu Chi Medical Foundation, Hualien, Taiwan; 4 Center for Innovation and Medical Education Research, Hualien Tzu Chi Hospital, Buddhist Tzu Chi Medical Foundation, Hualien, Taiwan; 5 Department of Education and Human Potentials Development, National Dong Hwa University, No. 1, Sec. 2, Da Hsueh Rd., Room C214-1, Shoufeng Township, Hualien County, 974301, Taiwan, 886 3-8903855

**Keywords:** generative AI, artificial intelligence, research integrity, reference verification, self-regulated learning, cognitive load theory, health professions education, medical education, faculty development

## Abstract

**Background:**

Generative AI can reduce the academic-writing burden on clinical health care professionals, but unsupervised use introduces citation hallucination (the confident fabrication or misattribution of references), which threatens research integrity. When a machine invents a source, it is termed “hallucination,” and when a person does it, it is termed “fabrication,” yet both are equally unacceptable. Existing health professions education writing workshops have rarely translated this concern into a concrete, reproducible source-verification procedure.

**Objective:**

This study aimed to describe the development and delivery of a 2-day workshop teaching the Atomic Sentence method, a source-anchored knowledge-modeling technique for AI-assisted literature synthesis, and evaluate its feasibility, acceptability, and short-term effect on research-idea development among clinical health care professionals.

**Methods:**

We conducted a single-cohort educational program evaluation of a 2-day workshop (≈16 contact hours) for 18 health care professionals across 4 campuses of a Buddhist medical network in Taiwan. Curriculum development used the ADDIE (Analysis, Design, Development, Implementation, and Evaluation) model; outcomes were framed with the Kirkpatrick model (levels 1‐2). The workshop implemented a published 7-step AI-assisted research workflow (topic exploration, literature search, knowledge-base management, reading, synthesis, writing, and peer-review simulation). Citation integrity was protected by confining citation generation to a source-grounded tool (NotebookLM) combined with atomic-sentence extraction and mandatory cross-reference verification; other, nongrounded tools supported discovery, reading, and writing. Feasibility was assessed by the workshop completion rate and the questionnaire response rate. Outcomes were an acceptability questionnaire covering 5 domains (usefulness of instructional materials, curriculum planning, time allocation, personal-goal attainment, and administrative support; each rated 0‐10) and a pre/post research-topic-transformation analysis (4 categories; 2 independent coders, Cohen κ); responses to an open-ended item on improvement suggestions were analyzed by qualitative content analysis. Reporting follows the GAMER (Guidance for AI Use in Medical Education Reporting) guidance for AI use in medical education.

**Results:**

All 18 (100%) participants completed the workshop and evaluation. Acceptability was high across all 5 domains (each median 10; overall median 10, IQR 9.3-10). The most variable domain was time allocation (range 5-10). Short-term improvement in the research topic (substantial transformation or refinement) occurred in 11 (61%; κ=0.77) participants; 2 (11%) had no structured question before or after. All 18 (100%) would recommend the workshop.

**Conclusions:**

A short workshop teaching the Atomic Sentence method was feasible and well accepted and was associated with short-term research-idea development in a multidisciplinary clinical cohort. Whether the method reduces citation hallucination or improves citation accuracy was not tested and requires controlled studies with objective outcomes, blinded raters, and longitudinal follow-up.

## Introduction

The expectation that clinical health care professionals engage in scholarly research has grown across health professions education (HPE) systems, yet the conditions enabling such engagement remain limited. Time scarcity, information overload, methodological unfamiliarity, and writing anxiety are well-documented barriers to research productivity among clinician-educators [1,2]. These barriers are often most acute for early-stage clinician-researchers who have a clinical observation but not yet a structured, testable question.

Generative artificial intelligence (GenAI) and large language models (LLMs) have emerged as potentially transformative tools for academic-writing support, offering rapid literature summarization and drafting, and a rapidly expanding body of research documents their applications in medical education [3]. However, citation hallucination (the generation of references that do not exist, or the misattribution of real references to claims they do not support) has been identified as a manifestation of broader LLM risks [4]. The wider ethical implications of LLMs in medicine, including bias, transparency, privacy, and a tendency to produce convincing but inaccurate content, have recently been examined in a systematic review [5]. Fabricated citations may even constitute research misconduct when they function as data [6]. Walters and Wilder [7] showed that ChatGPT fabricated bibliographic citations, and Gravel et al [8] reported fabricated references in responses to medical questions. In 1 evaluation of ChatGPT-generated medical content, only 7% of references were both authentic and accurate, with 47% fabricated and 46% authentic but bibliographically inaccurate [9]; across systematic-review tasks, reported hallucination rates ranged from 29% to 91% [10]. These failures are not confined to early chatbots: in a 2025 evaluation, GPT-4o fabricated roughly 1 in 5 citations outright and introduced bibliographic errors in nearly half of the remainder, performing worse on less-visible topics [11], and reference and DOI fabrication has been documented across research disciplines [12]. Fabrication of references by a machine is no less serious than fabrication by a person; we therefore treat hallucination as an integrity problem, not merely a writing artifact. Beyond outright fabrication, even genuine references are often cited with inaccurate bibliographic details [9], and misattribution, in which a real reference is cited for a claim it does not actually support, is a further, less well-quantified failure mode. In the clinical sciences, where reference accuracy underpins treatment recommendations and protocol adoption, such errors carry risks beyond bibliographic inaccuracy.

Two questions follow. First, why do clinicians rely on GenAI at all? In a Nature survey of more than 1600 researchers, faster data processing and time savings were among the most commonly reported benefits of AI tools, with improved grammar and style highlighted as a particular benefit of LLMs for those writing in a non-native language [13], and the time scarcity and limited familiarity with comprehensive literature searching that impede clinicians’ research productivity [1,2] make such assistance particularly attractive in clinical settings. Second, why is verification difficult? Awareness that hallucination can occur does not, by itself, give clinicians a workflow for preventing it. The International Committee of Medical Journal Editors (ICMJE) and the World Association of Medical Editors (WAME) require authors to disclose AI use, verify all AI-generated content, and assume full human responsibility for published material [14,15]. Yet systematic verification of references against their sources has never been routine in medical writing: a meta-analysis of quotation accuracy found errors in approximately 1 in 4 citations in medical journal articles (total quotation error rate 25.4%), long before GenAI [16]. In routine AI-assisted practice (which we define as prompting an LLM to expand notes into referenced prose and then lightly editing the result), this preexisting verification gap is compounded by the volume and fluency of machine-generated references. Few HPE writing workshops translate the ICMJE/WAME expectation into a teachable, reproducible procedure, and formal institutional policies and training for GenAI use in medical education remain uncommon [17].

To address this gap, we developed a 2-day workshop teaching the Atomic Sentence (AS) method, a source-anchored technique in which participants decompose literature into semantically complete, independently readable, source-traceable knowledge units before assembling AI-assisted drafts. By generating text only from author-supplied sources and verifying every sentence against its original, the method is designed to close the two pathways through which hallucination arises: fabrication (citing a nonexistent source) and misattribution (citing a real source for a claim it does not make). Crucially, because LLMs can fabricate content and citations even when explicitly instructed not to [7,8], the method places responsibility for verification with the author rather than with prompt-level instructions; the verification procedure itself is described in the Methods. The design draws on self-regulated learning [18,19] and cognitive load theory (CLT) [20]. This study addresses a question that necessarily precedes any test of the method’s effect on citation accuracy: whether a verification-centered workflow can be taught to busy clinical professionals in a short format that they will accept and complete. The aims were to (1) describe the development, delivery, and content of the workshop and the AS method in sufficient detail to permit replication; and (2) evaluate its feasibility, acceptability, and short-term effect on research-idea development among clinical health care professionals. We did not assess citation accuracy or hallucination reduction, which are the subject of planned controlled studies.

## Methods

### Study Design and Reporting

We conducted a single-cohort educational program evaluation using a postintervention questionnaire and a pre/post analysis of research-topic quality. This design supports Kirkpatrick Level 1 (reaction) and a proximal Level 2 (learning) indicator [21] and is appropriate for describing a novel educational program and its feasibility. Feasibility was operationalized as the proportion of enrolled participants who completed the 2-day workshop (completion rate) and the proportion who returned a completed evaluation questionnaire (response rate); acceptability and short-term research-idea development were assessed with the instruments described below. Because no comparison group was used, the study is descriptive. Reporting follows the GAMER (Guidance for AI Use in Medical Education Reporting) guidance for reporting AI use in medical education [22]; a completed checklist is provided as Checklist 1.

### Setting and Participants

Participants were recruited from 4 hospital campuses of the Tzu Chi Medical Network in Taiwan (Hualien, Taipei, Taichung, and Dalin) through an institutional email announcement circulated by each campus’s medical education office. The announcement described the program as a hands-on workshop on AI-assisted research writing, that is, learning to use AI tools to support literature reading, synthesis, and academic writing; it did not advertise hallucination prevention or research-topic development. Interested staff self-registered; 3 participants were nominated by their department supervisors. The 2-day workshop was held in Beitou, Taipei (December 13-14, 2025) and comprised approximately 16 contact hours. There was no prerequisite research experience and no exclusion criteria. Because this was a single offering of a capacity-limited, hands-on workshop, no formal sample-size calculation was performed; all participants who completed the workshop were included, consistent with a feasibility design.

### Theoretical Basis

Two theories informed the design. Self-regulated learning frames learning as a cycle of goal-setting, strategy monitoring, and self-evaluation [18,19]; the AS method structures these phases through PICO (Population, Intervention, Comparison, Outcome) goal-setting, source verification during reading, and self-assessment with the Semantic Purity Score (SPS). CLT distinguishes intrinsic, extraneous, and germane load [20,23]; externalizing literature into small, standardized units is intended to reduce extraneous load during synthesis. These theories guided design choices.

### The AS Method and Workshop Curriculum

#### Curriculum Development and Workflow Overview

**Figure 1.**
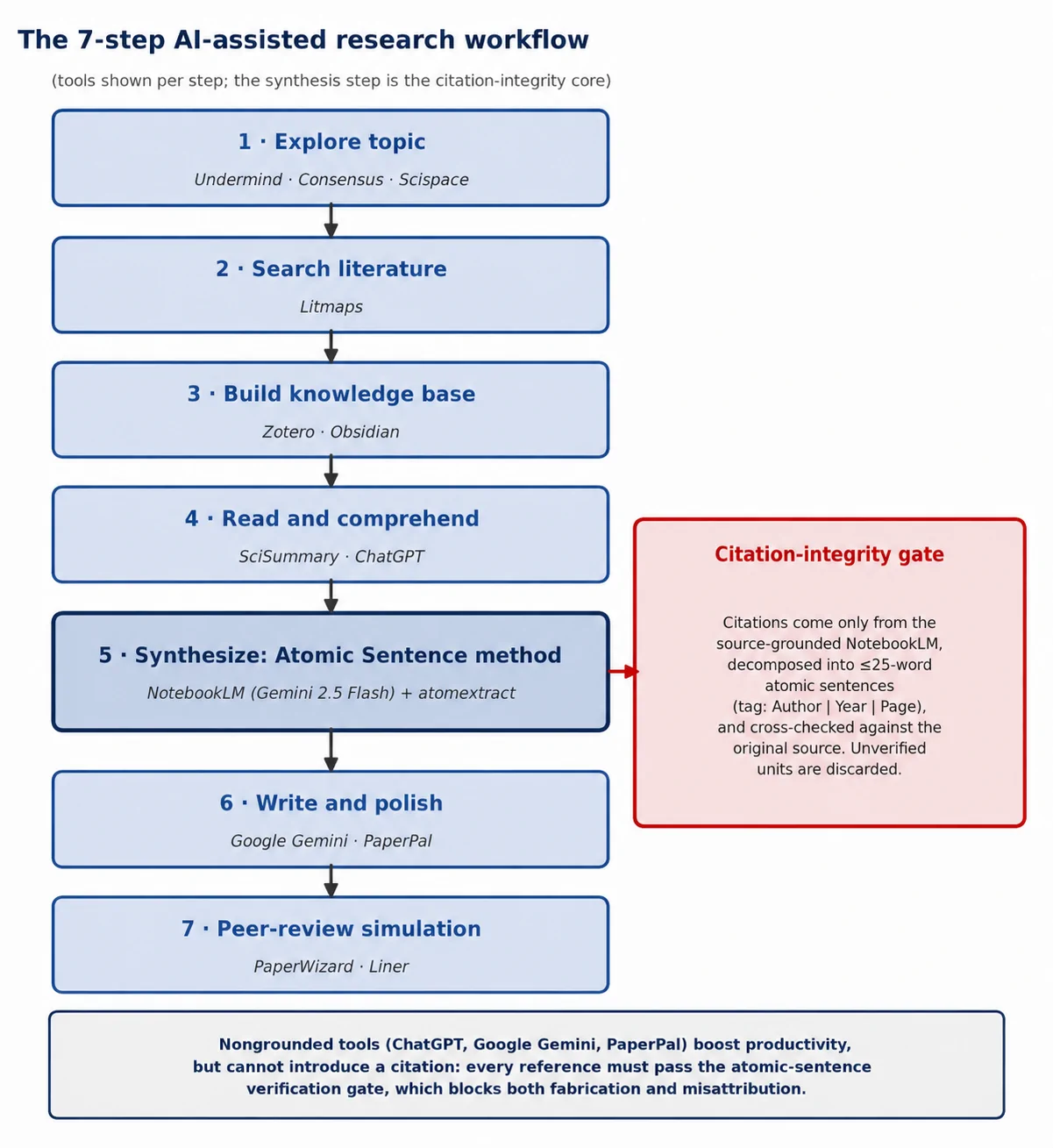
The 7-step AI-assisted research workflow taught in the workshop, with the tools used at each step. Citation integrity is protected at the synthesis step (highlighted): citations are generated only by the source-grounded tool NotebookLM, decomposed into atomic sentences and cross-checked against the original source, with any unit failing verification discarded. Nongrounded tools (eg, ChatGPT, Google Gemini, and PaperPal) support discovery, reading, and writing but cannot introduce a citation.

Curriculum development followed the ADDIE (Analysis, Design, Development, Implementation, and Evaluation) model [24]. Learning objectives progressed in line with the revised Bloom taxonomy, from understanding AI tool function, through analytic literature decomposition, to producing source-verified drafts [25]. The 4 stages are summarized in Table 1, and the workflow is shown in Figure 1. The curriculum operationalized a published 7-step AI-assisted research workflow [26]: topic exploration, literature search, knowledge-base management, reading and comprehension, synthesis, writing and polishing, and peer-review simulation. Each step used purpose-specific tools (Undermind, Consensus, and SciSpace for exploration; Litmaps for citation mapping; Zotero and Obsidian for the knowledge base; SciSummary and ChatGPT for reading; NotebookLM for synthesis; Google Gemini and PaperPal for writing and polishing; and PaperWizard and Liner for peer-review simulation). Critically, only the synthesis step generated citations, and it was confined to the source-grounded tool NotebookLM combined with the AS method and cross-reference verification (described below); the other tools, several of which are not source-grounded and can hallucinate, were used only for discovery, organization, reading, and prose editing, and any claim they produced still had to be reduced to a verified AS before it could enter a draft.

**Table 1.** The 4 workshop stages, activities, and corresponding learning objectives (revised Bloom level).

Stage	Activities	Learning objective (Bloom)
Research-question formulation	Articulate a clinical/educational observation; reformulate into a PICO^a^ question (Undermind, Consensus, SciSpace); facilitator modeling	Translate an observation into a testable question (Apply)
Literature discovery and gap mapping	Search and map papers (Litmaps); build a knowledge base (Zotero, Obsidian); read and summarize (SciSummary, ChatGPT); screen 8-12 papers; cross-check vs manual search	Locate and appraise relevant literature (Analyze)
Atomic Sentence method	Synthesis (NotebookLM+atomextract): decompose sources into ≤25 word, source-tagged units; self-score with SPS^b^; cross-reference verification; discard unverified units	Build verified, reusable knowledge units (Analyze/Evaluate)
Proposal drafting and AI disclosure	Write and polish (Google Gemini, PaperPal) an IMRaD^c^ proposal skeleton from verified units; peer-review simulation (PaperWizard, Liner); paragraph-by-paragraph source check; draft ICMJE^d^ AI disclosure	Produce a source-verified proposal draft (Create)

a PICO: Population, Intervention, Comparison, Outcome.

b SPS: Semantic Purity Score.

c IMRaD: Introduction, Methods, Results, and Discussion.

d ICMJE: International Committee of Medical Journal Editors.

#### Stage 1: Research-Question Formulation

Participants articulated a clinical or educational observation and iteratively reformulated it into a PICO question under facilitator guidance, while the facilitator modeled the transition from a vague narrative to a testable question. Initial topic exploration was supported by Undermind AI, Consensus, and SciSpace.

#### Stage 2: Literature Discovery, Screening, and Gap Mapping

Participants searched for and mapped key papers with Litmaps, organized them into a personal knowledge base with Zotero and Obsidian, and used SciSummary and ChatGPT to read and summarize candidate papers, assembling an initial set of 8 to 12 papers. Papers were included if they were peer-reviewed, directly relevant to the PICO question, and available in full text; the facilitator emphasized that AI assists but does not arbitrate literature quality. Participants read the candidate papers to judge quality, and all search records were retained and cross-checked against a manual database search.

#### Stage 3: The AS Method (Core)

Participants decomposed each source into semantically complete, independently readable, source-traceable knowledge units of ≤25 words. Each unit must satisfy 3 criteria: semantic completeness (understandable without surrounding text), independent readability (a colleague unfamiliar with the paper can accurately paraphrase it within 30 s), and recomposability (it can be combined with units from other sources to build new arguments). The ≤25 word limit keeps each unit within working memory limits and maximizes conciseness; in the source monograph, the highest-quality (9-point) exemplars are typically 18 to 22 words, whereas units exceeding approximately 25 to 30 words score lower and are split [27]. A central motivation for the method is that source-grounded tools used on their own often return only a general summary (the gist) rather than the specific, data-bearing claims needed for synthesis; by requiring semantic completeness (including key statistics) and a strict length limit, the AS method instead yields precise, independently citable knowledge units [27]. Each unit was tagged with metadata: Author | Year | Page | Functional role (Background/Method/Finding/Limitation). Quality was self-assessed using the SPS, a 9-point rubric spanning 5 dimensions (topic relevance, semantic completeness, independent readability, conciseness, and academic value); the full rubric and a worked example (paragraph → ASs → metadata → SPS scoring) are provided in Multimedia Appendix 1. This synthesis step is the integrity core of the workflow: atomic sentences were drafted with the aid of a purpose-built web tool (atomextract.web.app) that prompts the source-grounded tool NotebookLM (Google; powered by Gemini 2.5 Flash at the time of the workshop, December 2025 [28]) to draw candidate units strictly from the uploaded sources.

We define 2 terms used throughout. Source anchoring means generating content only from user-supplied source documents (here, via NotebookLM), so that outputs are tied to real, uploaded references. Cross-reference verification means manually checking each atomic sentence against the original PDF to confirm both that the reference exists and that it supports the stated claim.

Hallucination risk was addressed procedurally: (1) all citation-bearing content was generated only from the source-grounded tool (NotebookLM), and the nongrounded tools were restricted to noncitation tasks (search, reading, and prose editing); (2) every AS citing a specific statistic or claim was manually cross-checked against the original PDF before inclusion; (3) any unit failing verification was discarded rather than edited; and (4) each group’s facilitator manually reviewed participants’ atomic sentences and drafts, and participants presented their verified work to the cohort for peer and facilitator scrutiny. The number of discarded units was not recorded and no citation-accuracy rate was computed; these steps were formative parts of the teaching process.

#### Stage 4: Proposal Drafting and AI Disclosure

Using their verified atomic-sentence library, participants assembled an IMRaD (Introduction, Methods, Results, and Discussion) skeleton of a research proposal, defined here as a section-by-section outline for a study not yet conducted, not a manuscript reporting findings. Because no data had been collected, no Results were generated; the planned-analysis subsection described intended analyses only. Drafts were reviewed paragraph by paragraph against source documents, and a final check was performed for uncited references and for citations not supported by their sources. Participants used Google Gemini and PaperPal to draft and polish prose and PaperWizard and Liner to simulate peer review; because these tools are not source-grounded, no new citation could be introduced at this stage, and every reference had to originate from the verified atomic-sentence library. Each participant drafted an ICMJE-consistent AI-disclosure paragraph as a required deliverable. Representative prompt templates, which followed the published AS monograph, are listed in Multimedia Appendix 1.

### Evaluation Instruments

Acceptability was assessed with an anonymous, self-administered postworkshop questionnaire developed by the workshop faculty for internal quality evaluation (not previously validated). The questionnaire was administered via an online platform and was completed in person, on-site, at the end of the second workshop day. Five domains were rated from 0 (not at all) to 10 (extremely satisfied): (1) usefulness of the instructional materials and information; (2) overall curriculum planning; (3) adequacy of time allocation; (4) attainment of the participant’s personal goals; and (5) satisfaction with administrative and staff support. Two yes/no items asked about willingness to recommend the workshop and to attend future workshops, and 1 open-ended item invited improvement suggestions. Responses were collected anonymously.

Research-topic transformation was assessed by comparing verbatim responses to 2 open-ended items (“Describe your research topic before the workshop” and “...after the workshop”). Each participant was classified into one of four categories: (1) substantial transformation (vague or absent topic → PICO-structured, testable question); (2) refinement (an already-structured topic augmented with an explicit theoretical framework, comparison group, or measurable outcome); (3) retained (a structured topic that remained essentially unchanged); and (4) not yet formed (no structured question before or after).

### Data Analysis

Three analyses were performed; all are reported here. First, acceptability ratings: because of the small sample and the bounded, ceiling-skewed ratings, scores are summarized as the median (IQR) and range; means (SD) are also given in Table 2 for comparability. Percentages derived from study data are reported with their numerator and denominator and rounded to integers (with n=18, one participant is ≈6%). Second, research-topic transformation: 2 raters independently coded all 18 cases using the 4 category definitions; interrater agreement was quantified with the Cohen κ statistic, and disagreements were resolved by consensus. Third, responses to the open-ended item on improvement suggestions were analyzed by inductive qualitative content analysis [29]: 2 authors independently read all responses, condensed them into codes, and grouped the codes into descriptive categories, with disagreements resolved by discussion. Given the brevity of the responses, we report descriptive categories rather than interpretive themes. All 18 participants returned complete questionnaires; there were no missing data, and no imputation was required. Descriptive statistics were computed in Microsoft Excel and κ in Python.

**Table 2.** Postworkshop acceptability ratings by domain (N=18; 11-point scale, 0=not at all to 10=extremely satisfied).

Domain	Median (IQR)	Range	Mean (SD)
Usefulness of instructional materials/information	10 (10-10)	9-10	9.9 (0.2)
Overall curriculum planning	10 (10-10)	7-10	9.7 (0.8)
Attainment of personal goals	10 (9-10)	6-10	9.3 (1.3)
Adequacy of time allocation	10 (9-10)	5-10	9.2 (1.4)
Administrative and staff support	10 (10-10)	9-10	9.9 (0.2)
Overall (all domains)	10	N/A^a^	9.6

a N/A: not applicable.

### Ethical Considerations

This study was a retrospective analysis of deidentified evaluation data from an educational workshop held in December 2025 and was reviewed and approved by the Research Ethics Committee (REC) of Hualien Tzu Chi Hospital, Buddhist Tzu Chi Medical Foundation (IRB115-045-B; approved March 26, 2026). The REC reviewed the study as a retrospective educational program evaluation rather than interventional human subjects (clinical trial) research; it therefore did not fall under the provisions of the national human trial regulations governing ongoing clinical trials. Participants consented to the research use of their deidentified data, and the REC granted a waiver of documentation of written informed consent (the online questionnaire disclosed the research use of responses; voluntary completion implied consent). All data were anonymous and deidentified; no participant-identifiable information was collected, and verbatim topic descriptions were screened to remove any identifying details. No participant data were uploaded to any AI tool (only published literature sources were used with AI tools during the workshop), and no participant compensation was provided.

## Results

### Participants

All 18 (100%) enrolled professionals completed the 2-day workshop and returned the questionnaire. Their characteristics are summarized in Table 3. Physicians were the largest group (n=9, 50%), and most participants self-enrolled (n=15, 83%). Participant age and sex were not recorded.

**Table 3.** Characteristics of the 18 workshop participants by professional role, hospital campus, and enrollment route^a^.

Characteristic	Participants, n (%)
Professional role	
Physician	9 (50)
Nurse	6 (33)
Medical technologist	2 (11)
Administrative staff	1 (6)
Hospital campus	
Hualien	9 (50)
Dalin	5 (28)
Taipei	2 (11)
Taichung	1 (6)
Not specified	1 (6)
Enrollment route	
Self-enrolled	15 (83)
Supervisor-nominated	3 (17)

a Age and sex were not collected. Percentages use 18 as the denominator and are rounded to integers; therefore, they may not sum to 100% because of rounding.

### Feasibility and Acceptability

Completion and response rates of 18 (100%) support the feasibility of delivering the workshop across a multicampus network. Acceptability was high across all 5 domains (Table 2), with a median of 10 on every domain. The lowest and most variable ratings were for time allocation (range 5-10), consistent with the hands-on, time-intensive nature of source verification. All 18 (100%) participants reported willingness to recommend the workshop to colleagues and to attend future workshops.

### Research-Topic Transformation

Two coders independently classified all 18 participants; raw agreement was 15 (83%), and Cohen κ was 0.77 (substantial agreement). After consensus, short-term improvement in the research topic (substantial transformation [category A] or refinement [category B]) was observed in 11 (61%) participants: 6 (33%) had substantial transformation, and 5 (28%) had refinement. A total of 5 (28%) participants retained an already-structured topic (category C), and 2 (11%) had no structured question before or after (category D). Category counts are shown in Table 4. The A-versus-B boundary accounted for all coder disagreements, so we emphasize the combined “any improvement” figure as the more robust indicator.

**Table 4.** Pre/post research-topic transformation (N=18; 2 independent coders, Cohen κ=0.77)^a^.

Category	Definition	Participants, n (%)
A: Substantial transformation	Vague or absent topic → PICO^b^-structured, testable question	6 (33)
B: Refinement	Structured topic augmented with theory, comparison group, or measurable outcome	5 (28)
C: Retained	Pre-existing structured topic, essentially unchanged	5 (28)
D: Not yet formed	No structured question before or after the workshop	2 (11)
Any improvement (A+B)	Substantial transformation + refinement	11 (61)

a Percentages use 18 as the denominator. Coder disagreements occurred only at the A-versus-B (substantial vs refinement) boundary; the combined “any improvement” (A+B) figure is the more robust indicator.

b PICO: Population, Intervention, Comparison, Outcome.

A representative end-to-end transformation illustrates the process. One medical technologist entered with the topic “laboratory data analysis.” Through PICO formulation (Stage 1), source-anchored literature discovery and atomic-sentence synthesis (Stages 2-3), and proposal drafting (Stage 4), this became: “Is AI-based virtual quality-control teaching noninferior to conventional hands-on laboratory quality-control training for medical technologists’ competency?” A full paragraph-to-atomic-sentence worked example is provided in Multimedia Appendix 1.

### Improvement Suggestions

Of the 18 participants, 7 (39%) answered the open-ended item on improvement suggestions. Qualitative content analysis of these responses yielded 3 categories: preworkshop distribution of materials, more time for hands-on practice, and advance notice and preinstallation of required software. All 3 concerned session logistics rather than instructional content.

## Discussion

### Principal Findings

A 2-day workshop teaching the AS method was feasible to deliver across a multicampus clinical network and was highly acceptable, with uniformly high satisfaction and unanimous willingness to recommend it. Most participants showed short-term development of their research idea. Relative to the study aims, the workshop achieved its descriptive and feasibility objectives; it did not, and was not designed to, demonstrate a reduction in citation hallucination. Its contribution is to operationalize source verification as a concrete, teachable procedure rather than an abstract expectation.

### Comparison With Prior Work

Unlike workshops focused on framework selection or manuscript structure [30,31], this workshop foregrounds source-verified AI use and the earliest step of the research continuum: moving from a clinical observation to a testable question. The AS method adds an explicit quality rubric (the SPS) that makes unit quality visible during the session, enabling formative feedback, and specifies criteria precise enough to teach without domain-specific facilitator expertise. Our experience is consistent with the growing literature documenting frequent fabrication and misattribution in AI-generated citations [6-10] and with calls to translate ICMJE/WAME accountability mandates [14,15] into practice.

Citation inaccuracy did not begin with GenAI. Quotation errors affected roughly 1 in 4 citations in medical journal articles well before LLMs existed [16]; what GenAI changes is the scale, speed, and fluency with which unverifiable references can be produced [9,10]. The response of the research community has so far concentrated on disclosure and accountability: the ICMJE and WAME statements assign responsibility [14,15], and the GAMER statement asks authors to report how AI-generated content was verified [22], but none of these documents specifies how a busy clinician should actually perform verification. Surveys indicate that researchers adopt AI tools chiefly to save time [13], so a verification procedure that is itself slow and unstructured is unlikely to be used. Set against this literature, the workshop’s contribution is a procedure-level answer: it specifies who verifies (the author), against what (the original full text), at what granularity (the individual atomic sentence), and with what disposition rule (discard, never edit). We are not aware of other HPE curricula that make the verification step itself the central teachable unit rather than a closing exhortation.

### Implications

For HPE and faculty development, a short, structured workshop may help clinicians adopt a verifiable AI-writing workflow and may lower the threshold for initiating research by scaffolding the move from observation to question, an approach consistent with emerging practical guidance for integrating AI into medical education [32]. Unlike general exhortations to verify citations, the AS method makes verification structurally explicit: because each knowledge unit is generated only from an uploaded source and carries an author, year, and page tag, any claim that cannot be traced to a source is immediately visible and is discarded rather than edited. The method’s premise is that critical thinking cannot be delegated to the writing stage: fluent, plausible AI prose invites complacent acceptance, and meaning can drift even when no citation changes. The discard-not-edit rule and the paragraph-by-paragraph source review exist precisely to keep the author, not the model, responsible for every claim. The AS method is best positioned as an idea-development and integrity-supporting tool for early-stage clinician-researchers, rather than as a proven means of eliminating hallucination.

If workshops of this kind are subsequently shown to be effective, the practical benefits would extend beyond individual writing skills. Institutions would gain a shared, auditable vocabulary for AI-assisted writing (atomic sentences, SPS scores, verification records) that can be embedded in research-integrity training, journal clubs, and the supervision of early-stage clinician-researchers, and that gives mentors a concrete artifact to inspect rather than a finished draft to take on trust. The format is also inexpensive relative to its target problem: 2 days with commodity tools, set against the institutional cost of a single retraction or integrity investigation arising from fabricated references.

A broader implication concerns matching program design to participants’ entry readiness. That 7 of 18 participants did not improve their research topic is best read not as a shortfall of the workshop but as evidence that a single short format cannot serve a cohort of mixed readiness equally. For the 5 participants who entered with an already-structured question (category C), a ceiling effect is the most parsimonious explanation: the coding scheme registers change, and a well-formed PICO question leaves little room to improve; for these participants, the workshop functioned as synthesis and writing training rather than topic development. The 2 category D participants sit at the opposite end of the readiness spectrum: a 2-day format compresses topic formulation into hours, which may be too fast for staff who arrive without a candidate clinical observation. Read this way, the finding implies that programs teaching AI-assisted research skills should stratify participants by topic maturity and pair a short workshop with longitudinal support rather than treat it as self-sufficient. Concretely, this points to a brief preworkshop needs assessment, a preworkshop assignment to bring one concrete clinical observation, and a mentored topic clinic 1 to 3 months later for participants whose questions remain unformed. Feasibility and acceptability, which this study supports, are thus necessary but not sufficient conditions for uniform learning gains across a heterogeneous clinical workforce.

Whether the procedure changes downstream behavior (citation accuracy, completed proposals, or publications) remains to be tested.

### Limitations

This study has important limitations. First, and most importantly, we collected no objective measure of citation accuracy or hallucination; verification was facilitator-judged and not quantified, and the number of discarded units was not recorded, so the central premise of the method remains untested here. Second, the single-cohort, post-only design without a control group prevents causal or effectiveness claims. Third, 83% (15/18) of participants self-enrolled, so the sample was likely motivated, and the favorable ratings may be inflated; in addition, participants’ prior research experience, publication history, and familiarity with AI tools were not systematically recorded, and the recruitment announcement described an AI-assisted research-writing workshop, which will have attracted staff already interested in AI-assisted writing. Fourth, we performed no formal knowledge or skill assessment; research-topic transformation is a proximal level 2 indicator only, its category boundaries are partly subjective (κ reported), and it was assessed within a 48-hour window. Fifth, cognitive load was not measured, so CLT-based interpretations are conceptual. Sixth, the SPS and the AS method have had limited independent validation. Seventh, age and sex were not collected. Eighth, the sample (N=18) was small and drawn from a single network, limiting generalizability, and specific LLM versions and data-governance provisions were not fully standardized, limiting reproducibility. Finally, the corresponding author developed the AS method and authored the commercial monograph describing it and also led the workshop; this developer or instructor conflict of interest may bias design, delivery, and interpretation, and readers should weigh the findings accordingly.

### Conclusions

Teaching the AS method in a short workshop was feasible and well accepted and was associated with short-term research-idea development among clinical health care professionals. These exploratory findings do not establish that the method reduces citation hallucination or improves citation accuracy. Controlled studies with objective citation-accuracy outcomes, validated knowledge and skill assessments, blinded raters, and 3- to 6-month follow-up (eg, institutional review board submissions and manuscript completion) are needed to establish Kirkpatrick level 2-4 outcomes. The broader contribution of this work is a concrete, assessable vocabulary for AI source verification that other HPE programs can adapt.

## Supplementary material

10.2196/98343 Multimedia Appendix 1 The atomic sentence method.

10.2196/98343 Checklist 1 GAMER checklist.

## Abbreviations

ADDIE: Analysis, Design, Development, Implementation, Evaluation
AS: Atomic Sentence
CLT: cognitive load theory
GAMER: Guidance for AI Use in Medical Education Reporting
GenAI: generative artificial intelligence
HPE: health professions education
ICMJE: International Committee of Medical Journal Editors
IMRaD: Introduction, Methods, Results, and Discussion
LLM: large language model
PICO: Population, Intervention, Comparison, Outcome
REC: Research Ethics Committee
SPS: Semantic Purity Score
WAME: World Association of Medical Editors
