# Supplementary material for: Teaching the Atomic Sentence Method for Source-Verified, AI-Assisted Literature Synthesis to Clinical Health Care Professionals: Single-Cohort Feasibility and Acceptability Study
Source: JMIR Form Res. 2026 Aug 27;10:e98343. doi: 10.2196/98343 (PMC13521503; doi:10.2196/98343)
Supplement: Supplementary file 1 [file formative-v10-e98343-s001.docx]

**Multimedia Appendix 1**

**The Atomic Sentence Method: A Reproducible Procedure for Source-Verified, AI-Assisted Literature Synthesis, with the Semantic Purity Score Rubric, a Worked Example, and AI Prompt Templates**

# **1. Purpose and Scope**

This appendix provides an English-language, reproducible description of the Atomic Sentence (AS) method taught in the workshop, so that readers can apply and evaluate it without access to the Chinese-language source monograph. It specifies the verification workflow, the structure and quality criteria of an atomic sentence, the 9-point Semantic Purity Score (SPS) rubric, a fully worked decomposition example, the AI tools and prompt templates used, and the cross-reference verification procedure.

# **2. The Source-Verification Workflow**

The AS method chains four tool types so that every sentence used in a draft is traceable to a verified source. AI is used to accelerate discovery and decomposition; verification is performed manually.

**Step 1. Literature discovery.** Candidate papers are identified with AI discovery tools (Undermind AI; Litmaps) and screened for relevance to the PICO question, peer-review status, and full-text availability.

**Step 2. Source-grounded extraction.** Selected full-text PDFs are uploaded to NotebookLM (Google), powered by Gemini 2.5 Flash at the time of the workshop (December 2025), a source-grounded tool that answers only from uploaded documents. Atomic sentences are extracted using the prompt template in Section 7 (the atomextract.web.app helper can auto-generate this prompt).

**Step 3. Cross-reference verification.** Every atomic sentence that cites a statistic or claim is checked against the original PDF to confirm (a) the reference exists and (b) it actually supports the stated claim and number. Any sentence that fails is discarded, not edited.

**Step 4. Scoring, synthesis, and final check.** Each retained sentence is self-scored with the SPS; verified sentences are synthesized into prose (Section 7, Prompt 2) and reviewed paragraph-by-paragraph against sources, with a final check for uncited references and citations not supported by their sources.

# **3. The Atomic Sentence: Definition and Criteria**

An atomic sentence is a semantically complete, independently readable, source-traceable knowledge unit of ≤25 words. Each unit must satisfy three criteria:

1. **1) Semantic completeness**: it contains enough context (who, what, result, condition) to be understood without the surrounding text.
2. **2) Independent readability**: a colleague unfamiliar with the paper can accurately paraphrase it within 30 seconds.
3. **3) Recomposability**: it can be logically combined with units from other sources to construct new arguments.

Metadata format (appended to every unit): sentence | Author | Year | Page | #functional-tags

**Length guidance.** The ≤25-word target keeps each unit within working-memory limits and maximizes conciseness. By extraction intent: core findings 15–18 words; mechanism/theory 20–30; methods 25–35; limitations ≤25. Units exceeding ~25–30 words are split into separate atomic sentences.

# **4. The Semantic Purity Score (SPS)**

The SPS is a 9-point rubric across five dimensions, used for rapid self- and peer-assessment of each atomic sentence. A unit scoring <7 is revised or discarded.

| **Dimension (max)** | **Score = max** | **Score = 1** | **Score = 0** |
| --- | --- | --- | --- |
| Topic relevance (2) | Directly answers the core topic / key variable | Related but background or peripheral | Unrelated to the topic |
| Semantic completeness (2) | Who + what + result + condition; all key data explicit | Main message present; a minor detail missing (e.g., no CI) | Key information missing; original text required |
| Independent readability (2) | Fully understandable months later, without the original | Mostly clear; 1–2 concepts must be inferred | Pronoun-dependent; original text required |
| Conciseness (2) | Within target length; no filler words | Slightly over length (+5–10 words) or 1–2 fillers | Over ~40 words; redundant; needs major trimming |
| Academic value (1) | Contains quantitative or citable specifics worth reusing | n/a | No reusable, specific information |

**Total = 9.** Bands: 9 = ready to reuse without edits; 7–8 = high quality, minor fix; <7 = revise or discard.

# **5. Worked Example: Paragraph → Atomic Sentences**

***Source paragraph (illustrative).*** *“Smith et al. (2022) found that setting healthy meals as the default option in corporate cafeterias increased employees’ healthy-meal selection from 28% to 61% (N=1,247, p<.001). The effect was larger among younger employees (18–35; d=0.82) than older employees (≥55; d=0.31), remained stable across 12 weeks without novelty decay, and supports default options as a long-term workplace health-promotion strategy.”*

This single multi-claim paragraph is decomposed into four independently verifiable atomic sentences, each scored with the SPS:

| **#** | **Atomic sentence \| metadata** | **SPS** |
| --- | --- | --- |
| A (main effect) | In corporate cafeterias, setting healthy meals as the default raised employees’ healthy-meal selection from 28% to 61% (N=1,247, p<.001). \| Smith \| 2022 \| p.235 \| #default #dietary-behavior | 9 |
| B (moderator) | The default healthy-meal effect was larger in younger employees (18–35; d=0.82) than older employees (≥55; d=0.31). \| Smith \| 2022 \| p.236 \| #moderation #age | 9 |
| C (durability) | The default healthy-meal effect remained stable across 12 weeks with no novelty decay. \| Smith \| 2022 \| p.236 \| #durability | 9 |
| D (implication) | Sustained effectiveness of default healthy options supports their use as a long-term workplace health-promotion strategy. \| Smith \| 2022 \| p.237 \| #implication | 8 |

**Why decompose?** Each unit can be cited and recombined independently; a reader wishing to cite only the age-moderation finding need not quote the entire paragraph.

# **6. Worked Example: Detecting and Discarding Unverifiable Units**

Two failure modes are taught explicitly, because both are forms of citation hallucination:

**Fabrication / vagueness (discard).** “Research shows the nudge strategy has some effect on health promotion, but more research is needed. | source uncertain | p.?” = SPS 4 (vague, no data, unverifiable source). Cross-reference verification fails; the unit is discarded, not edited.

**Misattribution (discard).** A unit may carry a real, locatable citation (correct authors, year, and journal) yet state a claim the cited paper does not actually make. Because criterion (a) ‘the reference exists’ passes but criterion (b) ‘the reference supports the claim’ fails, the unit is discarded. Misattribution is the harder error to catch and is the main reason cross-reference verification requires opening the original PDF rather than only confirming the reference list.

# **7. AI Prompt Templates**

**Prompt 1. Atomic-sentence extraction (paste into NotebookLM after uploading source PDFs).**

```
[TASK] Precise atomic-sentence extraction from the uploaded source(s) ONLY.
[CORE TOPIC] <your PICO-aligned topic, e.g., "How does AI-based virtual
             quality-control teaching affect medical technologists' competency?">
[EXTRACTION INTENT] A1 = core findings (results, effect sizes, statistics)
   (A2 = mechanism/theory; A3 = methods; A4 = limitations)
[WORD LIMIT] A1: 15-18 words; A2: 20-30; A3: 25-35; A4: <=25
[QUALITY] semantically complete; independently readable; concise; SPS target >= 8
[GROUNDING] Use ONLY the uploaded sources. Do NOT add any fact not present in
   the sources. If a claim is not in the sources, omit it.
[OUTPUT] SimpleText, one per line:  sentence | Author | Year | Page | #tags
[RUN] Extract atomic sentences from the source(s) I have uploaded.
```

**Prompt 2. Synthesis (only AFTER manual verification).**

Organize the following VERIFIED atomic sentences into a coherent paragraph.
Preserve every citation exactly. Do NOT introduce any new claim or reference.
<paste verified atomic sentences here>

***Note.*** *Prompts follow the published AS monograph and are reproduced here in English. The atomextract.web.app helper can auto-generate Prompt 1 from the core topic and extraction intent.*

# **8. Cross-Reference Verification Procedure (Summary)**

**1.** Discover candidate literature (Undermind AI, Litmaps); screen for relevance, peer review, and full-text availability.

**2.** Upload selected PDFs to NotebookLM (source-grounded; Gemini 2.5 Flash at the time); extract atomic sentences with Prompt 1.

**3.** For each unit citing a statistic/claim, open the original PDF and confirm (a) the reference exists and (b) it supports the exact claim and number.

**4.** Discard (do not edit) any unit that fails verification.

**5.** Self-score each retained unit with the SPS; revise units <7 and split units >25–30 words.

**6.** Synthesize verified units (Prompt 2); review paragraph-by-paragraph against sources; final check for uncited references and unsupported citations.

# **9. AI Tools and Versions**

The workshop implemented a seven-step AI-assisted research workflow using: Undermind AI, Consensus, Scispace (topic exploration); Litmaps (citation mapping); Zotero, Obsidian (knowledge-base management); SciSummary, ChatGPT (reading); NotebookLM (Google; powered by Gemini 2.5 Flash at the time of the workshop, December 2025), the source-grounded tool used for citation generation; atomextract.web.app (atomic-sentence extraction helper); Google Gemini, PaperPal (writing and polishing); and PaperWizard, Liner (peer-review simulation). Only NotebookLM generated citations, and every cited claim was verified by cross-reference checking; the non-grounded tools (e.g., ChatGPT, Gemini, PaperPal) were used only for non-citation tasks and could not introduce a reference. All AI outputs were subject to the manual verification procedure above; the human authors take full responsibility for all content.
