# Supplementary material for: Teaching the Atomic Sentence Method for Source-Verified, AI-Assisted Literature Synthesis to Clinical Health Care Professionals: Single-Cohort Feasibility and Acceptability Study
Source: JMIR Form Res. 2026 Aug 27;10:e98343. doi: 10.2196/98343 (PMC13521503; doi:10.2196/98343)
Supplement: Supplementary file 2 [file formative-v10-e98343-s002.docx]

# Multimedia Appendix 2

GAMER Reporting Checklist (Reporting Guideline for the Use of Generative Artificial Intelligence Tools in Medical Research)

Reference: Luo X, Tham YC, Giuffrè M, et al; GAMER Working Group. Reporting guideline for the use of Generative Artificial intelligence tools in MEdical Research: the GAMER Statement. BMJ Evid Based Med. 2025;30(6):390-400. doi:10.1136/bmjebm-2025-113825.

| # | GAMER reporting item | How and where addressed in this manuscript |
| --- | --- | --- |
| 1 | General declaration of generative AI (GAI) use | Declared in the Methods (“Study Design and Reporting”) and in a dedicated “Generative AI Disclosure” section. GAI was used during the workshop (reported in the Methods) and in manuscript preparation: the manuscript’s reference list was prepared with the paper’s source-verification workflow (NotebookLM, with manual cross-checking of every citation), and Claude (Anthropic) and Gemini (Google) were used for English-language editing of author-written text. |
| 2 | GAI tool specifications (name, version, developer, settings) | Methods, Stages 2-4 and Multimedia Appendix 1, Section 9. Seven-step workflow: Undermind, Consensus, Scispace (exploration); Litmaps (mapping); Zotero, Obsidian (knowledge base); SciSummary, ChatGPT (reading); NotebookLM (Google; Gemini 2.5 Flash at the time) for source-grounded citation generation; atomextract.web.app (extraction helper); Google Gemini, PaperPal (writing); PaperWizard, Liner (peer-review simulation). Versions as available December 2025. Only NotebookLM generated citations; non-grounded tools were used for non-citation tasks. |
| 3 | Prompting techniques / prompts used | Representative prompt templates (4-element design) are provided verbatim in Multimedia Appendix 1, Section 7. |
| 4 | Role of the tool in the study | Methods, Stages 1-4: AI was used to discover literature, extract source-grounded atomic sentences, and assemble a proposal skeleton; all outputs were human-verified. AI did not generate study data, analysis, or conclusions. |
| 5 | Declaration of any new GAI model(s) developed | No new GAI model was developed. atomextract.web.app is a prompt-generation helper that operates existing tools (NotebookLM); it is not a trained model. |
| 6 | AI-assisted sections within the manuscript | Stated in the Generative AI Disclosure section: (1) reference-supported statements were drafted from the cited full texts with the source-grounded tool NotebookLM and every citation was manually cross-checked against its original source, with a final check for uncited references and unsupported citations; (2) Claude (Anthropic) and Gemini (Google) were used for English-language editing and rhetorical refinement of author-written text. No AI tool generated data, results, or conclusions. |
| 7 | Content verification | Methods, Stage 3 and Multimedia Appendix 1, Section 8: source-anchored generation; manual cross-reference verification of every cited statistic/claim against the original PDF; units failing verification were discarded, not edited; facilitator review and oral presentation. (Number of discarded units / a citation-accuracy rate were not quantified; this is stated as a limitation.) |
| 8 | Data privacy | Methods, “Ethical Considerations”: evaluation data were anonymous/de-identified. No patient- or participant-identifiable data were uploaded to any AI tool; only published literature sources were uploaded to NotebookLM during the workshop. |
| 9 | Impact on conclusions | Discussion: AI functioned as a taught aid within a human-verification workflow; the study’s feasibility/acceptability conclusions were derived by the authors, not by AI. Effectiveness against hallucination was not tested (Limitations). |

All GAMER reporting items are complete and consistent with the submitted manuscript.
